# Terrestrial venomous animals, the envenomings they cause, and treatment perspectives in the Middle East and North Africa

**DOI:** 10.1371/journal.pntd.0009880

**Published:** 2021-12-02

**Authors:** Timothy P. Jenkins, Shirin Ahmadi, Matyas A. Bittenbinder, Trenton K. Stewart, Dilber E. Akgun, Melissa Hale, Nafiseh N. Nasrabadi, Darian S. Wolff, Freek J. Vonk, Jeroen Kool, Andreas H. Laustsen

**Affiliations:** 1 Department of Biotechnology and Biomedicine, Technical University of Denmark, Kongens Lyngby, Denmark; 2 Naturalis Biodiversity Center, Leiden, the Netherlands; 3 Amsterdam Institute for Molecular and Life Sciences, Division of BioAnalytical Chemistry, Department of Chemistry and Pharmaceutical Sciences, Faculty of Sciences, Vrije Universiteit Amsterdam, Amsterdam, the Netherlands; 4 Centre for Analytical Sciences Amsterdam (CASA), Amsterdam, the Netherlands; 5 Department of Biomedical Engineering, Faculty of Engineering and Architecture, Eskişehir Osmangazi University, Eskişehir, Turkey; 6 Pharmaceutical Sciences Research Centre, Student Research Commitee, School of Pharmacy, Shahid Beheshti University of Medical Sciences, Tehran, Iran; 7 Department of Venomous Animals and Antivenom Production, Razi Vaccine, and Serum Research Institute, Karaj, Iran; Institut de Recherche pour le Développement, BENIN

## Abstract

The Middle East and Northern Africa, collectively known as the MENA region, are inhabited by a plethora of venomous animals that cause up to 420,000 bites and stings each year. To understand the resultant health burden and the key variables affecting it, this review describes the epidemiology of snake, scorpion, and spider envenomings primarily based on heterogenous hospital data in the MENA region and the pathologies associated with their venoms. In addition, we discuss the venom composition and the key medically relevant toxins of these venomous animals, and, finally, the antivenoms that are currently in use to counteract them. Unlike Asia and sub-Saharan Africa, scorpion stings are significantly more common (approximately 350,000 cases/year) than snakebites (approximately 70,000 cases/year) and present the most significant contributor to the overall health burden of envenomings, with spider bites being negligible. However, this review also indicates that there is a substantial lack of high-quality envenoming data available for the MENA region, rendering many of these estimates speculative. Our understanding of the venoms and the toxins they contain is also incomplete, but already presents clear trends. For instance, the majority of snake venoms contain snake venom metalloproteinases, while sodium channel–binding toxins and potassium channel–binding toxins are the scorpion toxins that cause most health-related challenges. There also currently exist a plethora of antivenoms, yet only few are clinically validated, and their high cost and limited availability present a substantial health challenge. Yet, some of the insights presented in this review might help direct future research and policy efforts toward the appropriate prioritization of efforts and aid the development of future therapeutic solutions, such as next-generation antivenoms.

## 1. Introduction

The total number of people that are medically affected by envenoming from snakes, scorpions, and spiders all around the globe runs into the millions. In the Middle East and North Africa (MENA), there are over 420,000 snakebites, scorpion stings, and spider bites each year [1,2]. The MENA region comprises 19 countries, i.e., Algeria, Bahrain, Egypt, Iran, Iraq, Israel, Jordan, Kuwait, Lebanon, Libya, Morocco, Oman, Palestine, Qatar, Saudi Arabia, Syria, Tunisia, United Arab Emirates, and Yemen. This region is home to over 30 species of venomous snakes, within the Elapidae, Viperidae, and Lamprophiidae families [1,2]. The MENA region is also home to 27 scorpion species across three families, i.e., Buthidae, Hemiscorpiidae, and Scorpionidae [3], as well as two medically important spider families, including widow spiders (*Latrodectus* spp.) and recluse spiders (*Loxosceles* spp.) [4]. Together, the venomous animals found in the MENA region present an impressive diversity of different venom toxins that can cause a range of local and systemic effects that can lead to permanent disability or death.

As is the case globally, the only effective treatment for severe venomous bites and stings in the MENA region is intravenous administration of antivenom. Antivenom is produced by immunizing large animals, such as horses and sheep, with sublethal doses of venom and then extracting and purifying the resulting polyclonal antibodies [5]. Although animal-derived antivenoms save numerous lives, many of these medicines suffer from several drawbacks, including a complicated and expensive production process, mismatch of demand–need–supply, poor quality, low specificity against small venom toxins, and often an inability to neutralize local tissue damage [6,7]. Fortunately, the rise of next-generation antivenoms based on defined recombinant cocktails of monoclonal antibodies, as well as recently investigated repurposed small molecule inhibitors and chelators, gives hope toward a substantial improvement in the treatment of envenoming victims in the near future [8–14].

However, to improve or replace current treatments and to effectively decrease the morbidity and mortality of envenomings in the MENA region, it is crucial to gain a better understanding of the magnitude of the problem and the variables involved. Therefore, in this review, we provide an overview of the epidemiology of snake, scorpion, and spider envenomings in the MENA region. Moreover, due to the relation between the venom composition and its pathophysiological effects [15], wherever possible/relevant, the key toxins of the venoms and their physiological effects are discussed. Addressing the biological activity of the venoms and their medically significant toxins not only helps to gain a comprehensive understanding of the envenomings, but also has a crucial impact on the design and development of new therapeutic approaches, such as next-generation antivenoms, to circumvent them [15,16]. Accordingly, the antivenoms that are currently in use in the MENA region to counteract animal envenomings and the future perspectives are discussed.

## 2. Main species responsible for severe envenomings

### 2.1. Snakes

**Fig 1:**
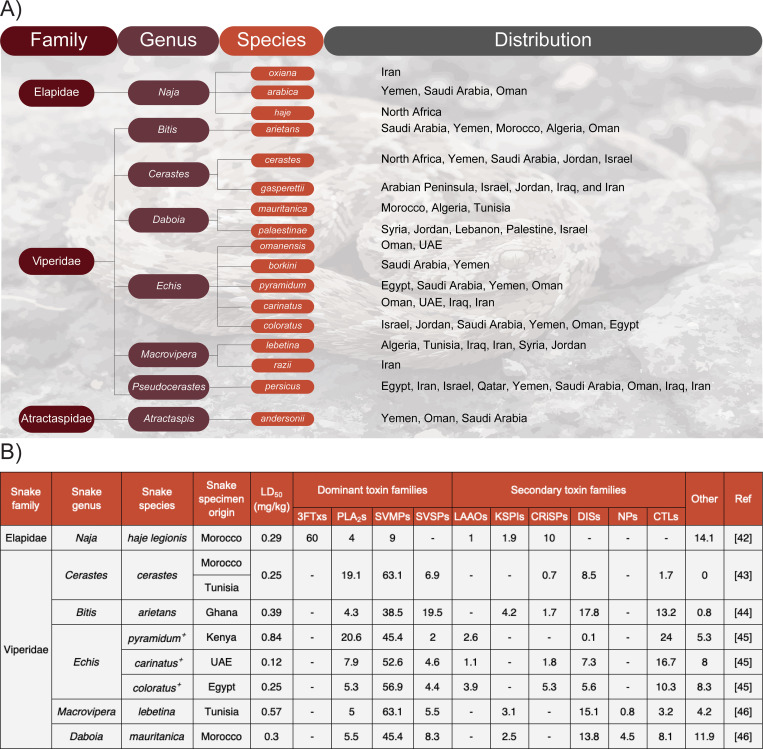
Medically relevant Category I snakes in North Africa and the Middle East. **(A)** Phylogeny and geographic distribution and **(B)** reported toxicity and available venom proteome or transcriptome (^+^) data are shown. Specifically, the table presents the genera, species, the origin of the reference proteome or transcriptome, and the median lethal dose in mice (LD_50_; mg/kg) [42–46]. It also illustrates the relative abundance of 3FTxs, PLA_2_s, SVMPs, SVSPs, LAAOs, KSPIs, CRiSPs, DISs, NPs, and CTLs. All components are represented as relative wet weight abundance in %. Notably, snake venom composition may vary depending on region. 3FTx, three-finger toxin; CRiSP, cysteine-rich secretory protein; CTL, C-type lectin; DIS, disintegrin; KSPI, kunitz-type serine protease inhibitor; LAAO, L-amino acid oxidase; NP, natriuretic peptide; PLA_2_s, phospholipase A_2_s; SVMP, snake venom metalloprotease; SVSP, snake venom serine protease.

There are over 731 different venomous snake species spread across the world (snakedb.org). However, the burden that these snakes cause to regions and communities is not evenly distributed. The highest burden is felt in sub-Saharan Africa and Southeast Asia, in part, due to the high number of highly venomous species found in these regions [17]. Consequently, these regions have so far received most of the international attention and resources. Conversely, the MENA region has been largely overlooked, despite numerous venomous species inhabiting this region. Indeed, according to the World Health Organization (WHO), a total of 16 Category I medically important snake species (i.e., highly venomous snakes that are prevalent in the respective country and cause numerous bites, leading to mortality or morbidity) are spread across North Africa and the Middle East (Fig 1A) [1]. Based on a literature review and modeling study performed by Kasturiratne and colleagues [17], North Africa was estimated to have between 463 and 36,208 snakebite incidences with a death toll between 20 and 29 individuals each year; the Middle East had similar figures, with 2,306 to 31,417 incidences and between 15 and 33 deaths annually. However, these statistics are very broad estimations seen from a global perspective and are expected to be significantly underestimated due to the lack of medical information in most of the assessed countries [17]. In the last 25 years, no reliable data involving snakebites exist from Algeria, Libya, Tunisia, Qatar, Syria, and Kuwait. This significantly skews the data for the whole MENA region and, in particular, North Africa, as only Egypt and Morocco are providing the epidemiological data for the entire region. As it remains, the actual number of incidences and deaths in this region will not be fully known until the lack of data from many countries is resolved. The available epidemiological data from the MENA countries reveal many similarities consistent across incidences involving snakebites. Numerous data reports show that most snakebite cases occur in rural farmable areas [18–24]. Specifically, multiple studies conducted in Egypt, Iran, Iraq, Morocco, and Saudi Arabia found that 70% or more of cases were reported from rural farming areas. Furthermore, males constitute more than 50% of snakebite incidences in many MENA countries [18,22,24–32]. Mortality rates from these countries range significantly and are largely dependent on the amount of available data. Epidemiological data from Egypt, Iran, Jordan, Morocco, Saudi Arabia, and Yemen have shown mortality rates ranging from 0.13% up to 4.8% [18,19,22–24,26,29,32]. Disturbingly, a study conducted on snakebite incidences in Iraq found a mortality rate of 7% to 15% among adults [27].

Responsible for this morbidity and mortality are the snakes’ toxins. Notably, while a great diversity in venom composition exists between and within different snake species, the key protein components that are present in these toxin cocktails often remain similar: Elapid venoms typically predominantly consist of three-finger toxins (3FTxs) and phospholipase A_2_s (PLA_2_s), whereas viper venoms often contain a substantial amount of snake venom metalloproteases (SVMPs), snake venom serine proteases (SVSPs), as well as PLA_2_s [7,33]. Currently, proteomics or transcriptomic data exist for half of the medically important snake species (nine out of 18), and based on that, SVMPs are the most prominent (average relative abundance of 41.9%) toxins in the MENA snakes, followed by C-type lectins (CTLs; 9.7%), PLA_2_s (9%), disintegrins (DISs; 8.5%), 3FTxs (7.5%), and SVSPs (6.4%; Fig 1B). SVMPs are important compounds of most viperid and crotalid venoms. The highest relative abundances of these toxins in the MENA snakes are found in the genera *Echis*, *Cerastes*, and *Macrovipera* (Fig 1B). They are known to cause hemorrhagic and local effects in victims through different mechanisms including disrupting extracellular matrix (ECM) of capillary endothelial cells, which, in turn, results into escaping of blood from capillaries. The second most abundant toxin family in the MENA snakes, i.e., snake venom CTLs (lectin-like proteins) [34]. CTLs are frequently found in the vipers and target clotting factors and various receptors on platelets, endothelial, and immune cells. CTL-related proteins can cause disseminated intravascular coagulopathies or severe bleedings, which both are life-threatening situations in viper envenomings [35]. Notably, PLA_2_s are another group of abundant toxins in MENA snakes, but their relative abundance varies greatly between different species, with the highest percentages observed in *Echis pyramidum* (20.6%) and *Cerastes cerastes* (19.1%) and the lowest in *Naja haje* (4%, Fig 1B). Besides affecting the blood coagulation cascade, PLA_2_s can have both neurotoxic and cytotoxic activities, resulting in systemic and local symptoms [36,37]. DISs constitute another group of abundant toxins in the MENA snakes and are frequently found in vipers and rattlesnakes and closely related to SVMPs. They are either produced from the proteolysis of a SVMP precursor or synthesized from mRNAs that have lost their metalloprotease-coding region [38]. DISs induce their toxicity through interacting with integrins on platelets, which are responsible for cell–ECM interactions and intercellular signaling [39], hence interfering with clot formation. Furthermore, while 3FTxs are only found in *Naja* species, they comprise more than half of their venom, e.g., *Naja nubiae* [40] and *N*. *haje* venom contains 70.9% and 60% of these toxins, respectively (Fig 1B). They are highly toxic and despite significant homology in sequence and structure, 3FTxs demonstrate a wide range of activities, from highly specific ion channel inhibition (neurotoxicity) to nonspecific membrane disruption (cytotoxicity). Finally, SVSPs are found in all viper species in the MENA region, with *Bitis arietans* presenting the highest relative abundance (19.5%; Fig 1B). In general, different SVSPs are able to both specifically activate the blood components that play a role in coagulation, fibrinolysis, and platelet aggregation (coagulant effect) and enzymatically degrade them (anticoagulant effect) [41].

The following presents an overview of all medically relevant snakes in the MENA region (see also [28]), their key toxins (Fig 1), and the role these toxins play in inducing envenoming symptoms.

#### 2.1.1. Elapidae family

Elapids have short, hollow, and fixed fangs [47], with venoms that are predominantly neurotoxic and characterized by progressive symptoms of paralysis and respiratory failure [48]. In the MENA countries, the Elapidae family is only represented by one genus, *Naja*, otherwise known as true cobras, that contains three species of Category I and one (*N*. *nubiae*) of Category II (potentially dangerous, but insufficient epidemiological data) medically important venomous snakes.

***Naja* genus.** The genus *Naja* contains over 30 species of highly venomous snakes [49], of which three have been identified within the MENA region. WHO considers *Naja arabica*, *N*. *haje*, and *Naja oxiana* as Category I venomous snakes. While *N*. *arabica* is found across Oman, Saudi Arabia, and Yemen, where it especially is a problem [1], *N*. *oxiana* is only located in Iran. Meanwhile, *N*. *haje* is distributed across North Africa and is known to be highly problematic in Algeria, Egypt, Libya, and Morocco (Fig 1A) [1].

Like many cobras, *N*. *haje* bites result in potentially lethal neurotoxic symptoms, as well as local swelling and pain at the bite site; these effects are primarily caused by neurotoxins (NTxs) and cytotoxins (CTxs) [42,50] from the 3FTx family. The NTxs mostly act by binding to the postsynaptic nicotinic acetylcholine receptors (nAChRs) at the neuromuscular junction to produce clinically significant and potentially lethal skeletal muscle paralysis [51]. Notably, in the MENA region, snake venom 3FTxs are only found in *Naja* species and have never been identified in the venoms of other MENA snakes. Yet, in the *Naja* genus, 3FTxs constitute a significant proportion of the whole venom and the majority of the venom’s toxicity. Indeed, proteomics data, which are only available for *N*. *haje*, reveals that 3FTxs (60%; Fig 1B) constitute the main venom component of these snakes. In addition to 3FTxs, PLA_2_s likely also play a role in inducing some of the *N*. *haje* envenoming symptoms. Indeed, PLA_2_s and CTxs from sub-Saharan African *Naja* species have been shown to act synergistically [52] and to be the primary components behind permanent tissue loss and the resultant amputations in *Naja* snakebite envenomings [53].

#### 2.1.2. Viperidae family

Besides elapids, another family of highly venomous snakes can be found in the MENA region, i.e., the family Viperidae, more commonly known as vipers. Vipers are characterized by their long, hollow, and retractable fangs [47] and often possess myotoxic and hemotoxic venom that can cause symptoms of local or systemic bleeding in the affected limbs, coagulopathy, and extensive edema [50]. This family also encompasses 12 of the 16 species identified by WHO as Category I snakes in the MENA region. These 12 species are spread across six genera, which are further discussed below.

***2*.*1*.*2*.*1*. *Bitis*.** The *Bitis* genus is widespread across the African continent and the southern Arabian Peninsula and consists of 17 species [49]. However, only one species, *B*. *arietans*, can be found within the MENA region. This species is known to inhabit Algeria and Morocco, but its distribution also extends to the Arabian Peninsula, including Oman, Saudi Arabia, and Yemen (Fig 1A) [49]. A study conducted by Eljaoudi and colleagues [50] found that *B*. *arietans* is responsible for approximately 17% of all recorded snakebites in Morocco. Notably, the large amounts of venom injected during an average bite (typically between 150 and 350 mg), alongside its toxicity, renders this species highly dangerous [54]. The venom of *B*. *arietans* can induce severe local and systemic clinical manifestations, including bleeding, swelling, necrosis, compartment syndrome, and hypotension [54]. Furthermore, based on proteomics data, *B*. *arietans* possesses the highest relative abundance (19.5%; Fig 1B) of SVSPs among all MENA snakes. SVSPs can generally be found in viper venoms and are known to affect the blood coagulation cascade at different levels, resulting in the inactivation of blood clotting factors, inhibition of platelet aggregation, and a decrease in blood pressure by acting as a hypotensive agent [42,46,55]. Besides SVSPs, CTLs make up a significant amount of *Bitis* venoms. These toxins (e.g., bitiscetin) are hemostasis disruptive and consequently prevent blood clotting by interfering with platelet aggregation and the coagulation cascade [35].

***2*.*1*.*2*.*2*. *Cerastes*.** One of the most widely distributed viper genera across the Sahara and Arabian Peninsula is the genus *Cerastes*, which consists of only three distinct species [56]. Two, i.e., *C*. *cerastes* and *Cerastes gasperettii*, are considered medically relevant. These species are dispersed across the MENA region (Fig 1A) and are responsible for many snakebite incidences. In particular, a study conducted by Al-Sadoon over the period from 2005 to 2010 found that in the Riyadh Province of Saudi Arabia, *C*. *gasperettii* was responsible for 886 snakebite cases, accounting for 86.9% of all incidences in this area [32]. In southern Morocco, *C*. *cerastes* is considered the most dangerous snake species [57].

A *C*. *gasperetti* envenoming causes local pain, swelling, edema, and tenderness in most patients [32]. Similarly, the danger from *C*. *cerastes* can be attributed to its venom causing mild to severe local effects, hypotension, and arterial thrombosis [50,58]. Currently, proteomics data are only available for *C*. *cerastes*; this suggests that SVMPs and PLA_2_s are the main constituents of its venom. SVMPs are capable of damaging ECM components, such as proteoglycans [59], collagen, laminin, and fibronectin [60]. Thus, SVMPs are mainly involved in edema, inflammation, swelling, blistering, skin damage, tissue necrosis, myonecrosis, and cardiovascular shock. In addition, they have procoagulant, anticoagulant, and platelet aggregation inhibitory effects [60]. In the MENA region, *Cerastes*, together with *Echis* and *Macrovipera* (see Sections 2.1.2.4, 2.1.2.5, and Fig 1B), possesses the highest relative abundances of SVMPs.

***2*.*1*.*2*.*3*. *Daboia*.** Like *Cerastes*, the genus *Daboia* only contains two medically relevant species within the MENA region, *Daboia mauritanica* and *Daboia palaestinae*. The former can be found within Algeria, Morocco, and Tunisia, whereas the latter is distributed across Israel, Jordan, Lebanon, Palestine, and Syria (Fig 1A). *D*. *mauritanica*, in particular, has been suggested to be responsible for most of the snakebite incidences within North Africa [49]. This species was linked to 69.4% of all the snakebite incidences that Eljaoudi and colleagues identified in Morocco [50]. Epidemiological data are scarce for *D*. *palaestinae*, but it has been reported that bites are associated with a death rate of 6.2% in Israel and Jordan [49].

Notably, the venom of *D*. *palaestinae* is cytotoxic and can induce hematuria, swelling, bleeding at the site of the bite, and significant hemorrhage [49]. Similarly, the venom of D. *mauritanica* is primarily cytotoxic and is characterized by mild to severe local effects, hypotension, and coagulopathy [50]. Proteomics data are only available for *D*. *mauritanica* (Fig 1B) and demonstrates that its venom predominantly contains SVSPs and DISs that inhibit platelet aggregation and integrin-dependent cell adhesion.

***2*.*1*.*2*.*4*. *Echis*.** *Echis* constitutes another genus of vipers in the MENA region and is one of the most taxonomically problematic Viperidae genera. The number of currently recognized species ranges from 8 to 12, and potentially 20 subspecies, depending on the literature [61]. Five of these species can be found in the MENA countries: *Echis burkini*, *Echis carinatus*, and *Echis omanensis*, which are predominantly located across the Middle Eastern countries, as well as *E*. *pyramidum* and *Echis coloratus*, which are mainly found in Egypt but also in the ME (Fig 1A). Indeed, a bite from this genus, without antivenom treatment, has an estimated mortality rate of up to 20% [45]. *E*. *coloratus*, in particular, is one of the key species contributing to this high mortality rate. It was identified as the primary species causing snakebite-related deaths in two different studies in Saudi Arabia. The first study found that *E*. *coloratus* accounted for 67% of all the Riyadh Province’s identifiable snakebite cases [18], yet this statistic did not hold true in a later study that demonstrated that *C*. *gasperettii* was the primary cause of death. The second one focused on snakebites among children in the Al-Baha region and found that this species accounted for 10.8% of the identifiable cases [20]. Furthermore, *E*. *carinatus* was responsible for up to 9% of snakebites in Oman, and alongside *E*. *omanensis*, it is thought to be the primary cause for snakebites in this country [31]. *E*. *carinatus* has also been identified as the species which causes highest mortality and morbidities in Iran [22].

General symptoms of *Echis* bites include local swelling at the bite site, local blistering, necrosis, incoagulable blood, and spontaneous systemic bleeding. The proteomics data available for *E*. *carinatus*, *E*. *coloratus*, and *E*. *pyramidum* offer a possible reason. Venom from these snakes predominantly contains SVMPs and CTLs. Besides local effects, SVMPs together with CTLs, have anticoagulant and platelet-modulating activities [62].

***2*.*1*.*2*.*5*. *Macrovipera*.** The genus *Macrovipera*, a smaller and taxonomically simpler genus than *Echis*, currently contains just three species and five subspecies [63]. *Macrovipera lebetina* is the most prominent species from this genus, with an extensive geographic range from the Middle East extending throughout central Asia (Fig 1A) [64,65]. In addition, it is the only species from this genus that is considered as a Category I medically important species by WHO. On the Iranian plateau (i.e., Afghanistan, Azerbaijan, Iran, Iraq, Pakistan, and Turkmenistan), this species is considered one of the most venomous snakes [64]. It is noteworthy that *Macrovipera razii* has recently been separated from *M*. *lebetina* as a new species that is endemic to Iran; however, its epidemiologic data are still not available [63].

The venom of *M*. *lebetina* is known to cause significant local tissue damage, necrosis, edema, and hemorrhage [64]. Nevertheless, the primary cause of death by this species has been attributed to acute renal failure [64]. The underlying toxin(s) that induce acute renal failure have not been identified. However, the venom of *M*. *lebetina* was found to be dominated by SVMPs (Fig 1B) that have been hypothesized to cause nephrotoxicity through secondary effects, including hypotension, hemolysis, and rhabdomyolysis [64].

***2*.*1*.*2*.*6*. *Pseudocerastes*.** Finally, the genus *Pseudocerastes* was given its name due to its phenotypic similarity to the *Cerastes* genus, despite a lack of genetic relatedness. *Pseudocerastes* contains three species with a geographical distribution ranging from Egypt to Iran and extending to the southeastern part of the Arabian Peninsula (Fig 1A) [49]. Nonetheless, *Pseudocerastes persicus* is the only clinically relevant species recognized within the MENA region. This species’ venom is thought to be highly coagulopathic, but little is known about its symptomatic effects in humans [66]. *Pseudocerastes fieldi* inhabits a similar geographic range as *P*. *persicus* and is classified as a Category II species by WHO. Notably, its venom substantially differs from *P*. *persicus* and is thought to possess neurotoxic properties [66]. This neurotoxicity is attributed to a novel PLA_2_ complex that creates an irreversible neuromuscular blockade at the presynaptic site [66].

#### 2.1.3. Lamprophiidae

The last of the represented snake families, Lamprophiidae, contains a single subfamily that is regarded as medically relevant in the MENA region. The species in the Atractaspidinae subfamily are known for their unique jaw that allows a single fang to protrude from an almost fully closed mouth [67].

This subfamily’s venom is cardiotoxic, with general symptoms of mild to moderate pain, swelling, and local discoloration [67]. However, this venom has also been associated with producing lethal coronary vasospasms, i.e., intense vasoconstriction of the coronary artery [68].

***Atractaspis*.** The genus *Atractaspis* contains 22 different species, which are distributed across Africa and the Middle East, but only two, i.e., *Atractaspis engaddensis* and *Atractaspis andersonii*, are found within the MENA countries [69]. Envenomings have been associated with anaphylactic shock, coronary vasoconstriction, and local necrosis [70]. Additionally, only *A*. *andersonii*, found in Oman, Saudi Arabia, and Yemen, is considered a Category I species. Clinical data are usually scarce for Category II snakes, yet three deaths due to *A*. *engaddensis* envenoming have been reported from Saudi Arabia. The deaths involved two children and one 30-year-old adult who all died within 1 hour from being bitten as a result of severe coronary vasospasm [71].

In humans envenomed by *A*. *engaddensis*, local effects are observed within minutes and are shortly followed by systemic effects of vomiting, weakness, hypoxia, liver damage, skin necrosis, and possibly fatal cardiac conduction disorders [72,73]. Species belonging to the *Atractaspis* genus are the only snakes known to produce another toxin family containing strong vasoconstrictor isopeptides, i.e., sarafotoxins (SRTXs) [72]. This toxin constitutes around 18% of *Atractaspis microlepidota engaddensis* venom [73] and is considered to be one of the most potent vasoconstrictors described to date and is stipulated to be the primary cause of death in victims [74].

### 2.2. Scorpions

Compared to sub-Saharan Africa and Asia, snakebite morbidity and mortality are relatively low in the MENA region. Conversely, incidences of accidental envenoming of humans by dangerous scorpions (scorpionism) and the resultant lethality are higher than anywhere else in the world. Global estimates surpass 1.2 million cases of scorpion envenoming and 3,250 deaths each year. The number of stings in the ME and NA has been estimated to be around 146,000 and 350,000, respectively, which, together, make up 42% of the global scorpion sting burden. The morbidity and mortality rates, although not accurate due to inaccurate/scarce data, are presented as 0.42% (613) and 0.55% (803) for the ME and 0.52% (1,820) and 0.23% (805) for NA, respectively [75]; notably, since the last available study is from 2008, these numbers might differ significantly today. Compared to other regions of the world, the mortality rate for ME and the morbidity rate for NA are the highest worldwide.

Among the 19 countries of the MENA region, Algeria, Egypt, Iran, Israel, Morocco, Saudi Arabia, and Tunisia are particularly affected by scorpionism [76]. For instance, in Tunisia, 40,000 stings are reported annually, with a mortality rate of 7.5% in the southern part of the country [77,78]. In addition, 12 different Buthidae family species in Morocco are responsible for an average of 25,000 stings every year, with a lethality rate of 0.40% (100) [79,80]. In Algeria, six species of this family are found, which cause 50,000 stings with a lethality rate of 0.22% (110) each year [81].

**Fig 2:**
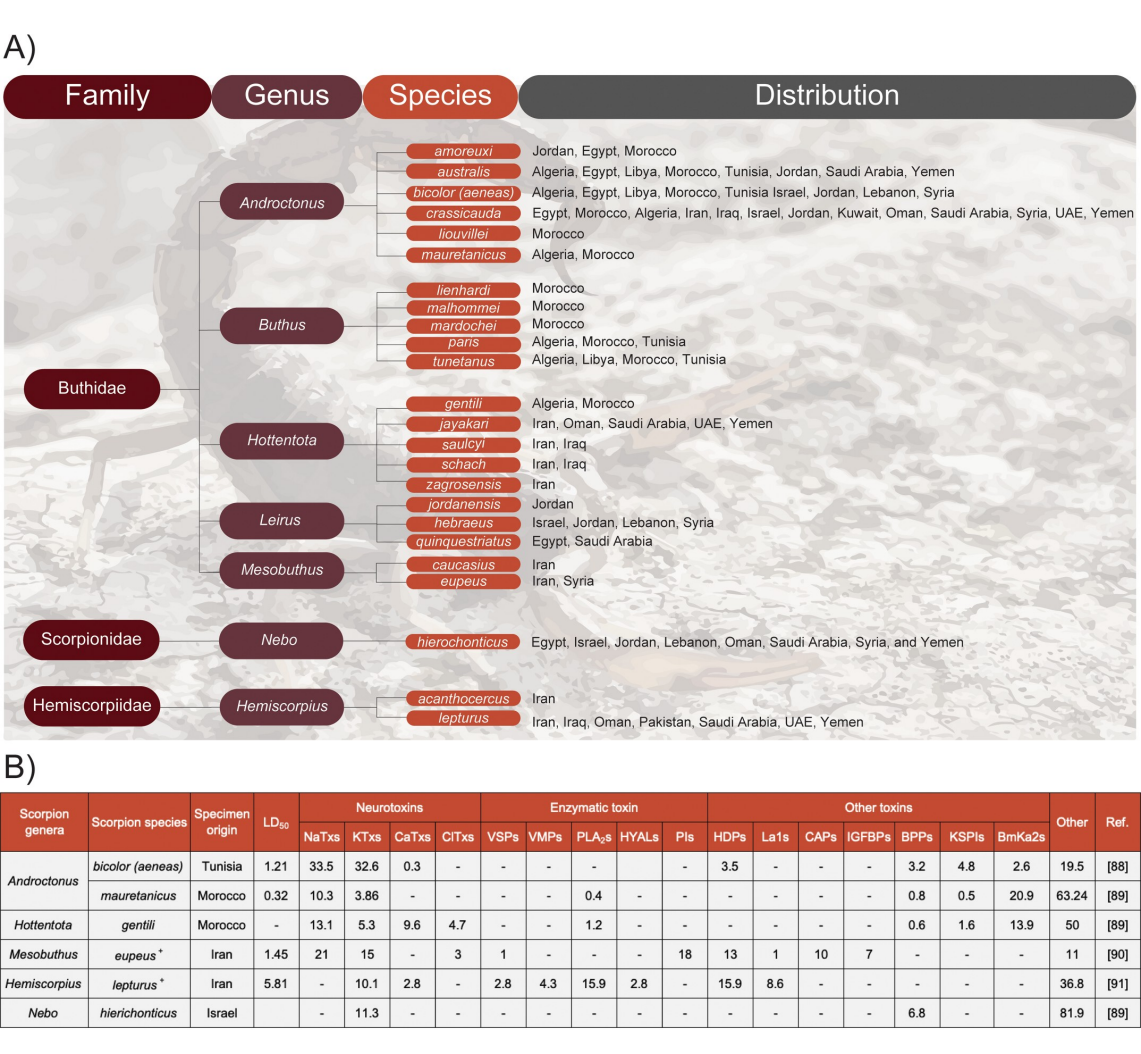
Medically important scorpions in North Africa and the Middle East. **(A)** Phylogeny and geographic distribution and **(B)** reported toxicity and available venom proteome or transcriptome (^+^) data are shown [88–91]. Specifically, the table presents the genera, species, the origin of the reference proteome or transcriptome, and the median lethal dose in mice (LD_50_; mg/kg). It also illustrates the relative abundance of toxins acting on Na^+^ channels (NaTx), toxins acting on K^+^ channels (KTxs), toxins acting on Ca^2+^ channels (CaTxs), toxins acting on Cl^−^ channels (ClTxs), VSPs, VMPs, PLA_2_s, HYALs, PIs, HDPs, La1s, antigen-5 and CAPs, IGFBPs, BPPs, KSPIs, NDBPs (e.g., BmKa2s), and other (often a result of lacking data). All components are represented as relative wet weight abundance in %. Notably, scorpion venom composition may vary depending on region. BPP, bradykinin-potentiating peptide; CAP, pathogenesis-related protein; HDP, host defense peptide; HYAL, hyaluronidase; IGFBP, insulin-like growth factor binding protein; KSPI, kunitz-type serine protease inhibitor; La1, La1 peptide; NDBP, non-disulfide-bridged peptide; PI, protease inhibitor; PLA_2_, phospholipase A_2_; VMP, venom metalloproteinase; VSP, venom serine protease.

Among the 2,200 scorpion species described to date, only a small percentage of them are known to be dangerous to humans. In the MENA region, most stings and hospitalizations can be attributed to the scorpion species belonging to *Androctonus*, *Buthus*, *Hottentotta*, *Leiurus*, and *Mesobuthus* genera of the Buthidae family and *Hemiscorpius* genus of the Hemiscorpiidae family (Fig 2A).

The Buthidae family, as the largest scorpion family, comprises 86 genera and over 900 species [82]. It is also the most widespread family of scorpions [82], occupying all six faunal regions of the world. Therefore, it is not surprising that Buthidae is the main family of medically important scorpions all around the world, including the MENA region, where 36 out of 101 venomous species of this family can be found [83]. While all of the medically important scorpion species in other parts of the world belong only to the Buthidae family, the three exceptional non-Buthidae scorpion species with medical importance can be found in the MENA region. These three species are members of two families, namely Hemiscorpiidae and Scorpionidae. The most venomous non-Buthidae family of scorpions is Hemiscorpiidae, which consists of one genus (*Hemiscorpius*) and 15 species [84]; two of these species are medically important (see Section 2.2.2). The family Scorpionidae contains some of the world’s largest living scorpions (up to 20 cm in length) distributed across seven genera [82]. The only medically important species of this family belongs to the genus *Nebo* (see Section 2.2.3).

In comparison to snakes, scorpion venoms are less complex. In fact, the most clinically relevant effects of scorpion venoms are caused by the inhibitory actions of two types of NTxs, namely NaTxs (Na^+^ channel toxins) and KTxs (K^+^ channel toxins) [85]. These two toxins also have the highest relative abundance in the MENA scorpions (average of 14.1%, ranging between 0% and 33.5%, and 13%, ranging between 5.3% and 32.6%, respectively; Fig 2B). These toxins, unlike snake NTxs, affect the voltage-gated Na^+^- and K^+^-channels, thus interfering with the generation and propagation of action potentials in neurons and resulting in potentially lethal paralysis in humans [85,86]. Unfortunately, to date, proteomics or transcriptomic data exist for only seven out of the 27 scorpion species (Fig 2B) [87].

An overview of the medically relevant scorpions in the MENA region and their most important toxins is presented below (Fig 2).

#### 2.2.1. Buthidae

***2*.*2*.*1*.*1*. *Androctonus*.** Currently, the genus *Androctonus* (fat-tailed scorpions) comprises 22 species [92], many of which can be found in African countries north of the Equator, as well as in the ME and all the way to India [93]. However, out of the 22, only six species, namely *Androctonus liouvillei*, *Androctonus amoreuxi*, *Androctonus bicolor* (previously *Androctonus aeneas*), *Androctonus mauretanicus*, *Androctonus australis*, and *Androctonus crassicauda* are considered medically important [83].

*A*. *liouvillei* is only found in Morocco and is responsible for 9% of stings and 14% of deaths in the country’s southwestern part [94]. Furthermore, *A*. *amoreuxi* shares the same geographical range and is responsible for approximately 17% of scorpion stings in southern Morocco [94], but its distribution also stretches to Egypt and Jordan. Another medically important species, *A*. *bicolor*, is distributed across a wide geographical range from NA to the ME (as far as Morocco in the west and Syria in the east) [95]. However, human contact with this scorpion appears to be rare [78]. Indeed, in Tunisia, during 2018, only seven cases of *A*. *bicolor* stings were recorded [96]. Conversely, *A*. *australis* and *A*. *mauretanicus* are responsible for most envenoming cases in Tunisia (86.7%) [96] and 13% of annual lethality cases in the southeast of Morocco, respectively [97]. Stings from *A*. *mauretanicus* can cause clinical manifestations such as respiratory disorders, including tachypnea, acute pulmonary edema, thrombocytosis, hyperglycemia, as well as hepatic and renal damage [97]. However, lethality is primarily associated with altering the mode of action in sodium and potassium channels via NaTxs and KTxs (Fig 2B) [98]. These toxins are also responsible for lethality caused by *A*. *crassicauda*, which is unsurprising since its venom profile is very similar to that of *A*. *australis* and *A*. *mauretanicus* [83]. Notably, *A*. *crassicauda* has the most prevalent distribution in the MENA region, spanning from Morocco to the Arabian Peninsula. However, its distribution also expands outside of the MENA region and also includes Armenia, Azerbaijan, and Turkey [83]. Furthermore, *A*. *crassicauda* is one of the two most dangerous scorpion species in Egypt, the other one being *Leiurus quinquestriatus* (see Section 2.2.1.3). These two species represent all the reported scorpion stings in Egypt (about half each) [99]. *A*. *crassicauda* is the most abundant and one of the most dangerous species among seven other medically important scorpion species in Iran [100]. Similarly, in Saudi Arabia, with 224 incidences per 100,000 people, the highest morbidity rate due to scorpionism is attributed to *A*. *crassicauda* [101], emphasizing the medical importance of this species all over the MENA region.

***2*.*2*.*1*.*2*. *Buthus*.** *Buthus* is the fourth largest genus of the Buthidae family, with 52 species widely distributed throughout Europe, Africa, and Asia [102]. Due to a significant increase in the description of new *Buthus* species over the past 15 years, the taxonomy of this genus has dramatically changed. For instance, *Buthus occitanus*, which once was reported as the most medically important species of the genus, causing severe/lethal envenomings in Morocco, now has been limited to France and Spain, while 17 other *Buthus* species have been assigned to Morocco [102]. As a consequence, the true epidemiology of the different species of this genus and the health burden they cause remain elusive. Nevertheless, at least five species of this genus, i.e., *Buthus lienhardi*, *Buthus malhommei*, *Buthus mardochei*, *Buthus paris*, and *Buthus tunetanus*, are considered to be medically important species [83].

***2*.*2*.*1*.*3*. *Hottentotta*.** Similar to *Androctonus* and *Buthus*, *Hottentotta* is a large genus. Formerly known as *Buthotus*, the *Hottentotta* genus is currently divided into 27 species, which are spread across Africa, the ME, and India [103]. In the MENA region, *Hottentotta khoozestanus*, *Hottentotta zagrosensis*, *Hottentotta saulcyi*, *Hottentotta schach*, *Hottentotta jayakari*, and *Hottentotta gentili* are the main species that may pose a medical threat to humans. While *H*. *khoozestanus* and *H*. *zagrosensis* are endemic to Iran, *H*. *saulcyi* and *H*. *schach* are found both in Iran and Iraq, and *H*. *jayakari* is distributed across the whole Arabian Peninsula, including Oman, Saudi Arabia, UAE, and Yemen. Notably, *H*. *gentili* is the only species of this genus found in NA, specifically in Algeria and Morocco [103]. In 2001, an epidemiological survey based in the southwest of Morocco reported that *H*. *gentili* was responsible for 14% of stings (130 out of 912) and 26% of deaths (nine out of 35) [104]. Similarly, A 12-year epidemiologic study carried out between 1994 and 2006 reported 724 envenomings and 32 deaths in the same country; *H*. *gentili* was responsible for 11% of the stings and 32% of the deaths, making it the second-most important species in the studied area [105]. Partial characterization of *H*. *gentili* venom has revealed that it contains all four types of NTxs, including NaTxs, KTxs, CaTxs (calcium channel toxins), and ClTxs (chloride binding toxins; Fig 2B). Thus, it is likely that severe localized pain that is prevalent in envenomings caused by the *Hottentotta* genus is mediated by NTxs [106–108]. In addition to localized pain, severe sympathetic symptoms, such as tachypnea, convulsions, and parasympathetic symptoms with potentially fatal outcomes, have been reported for this genus [94,106].

***2*.*2*.*1*.*4*. *Leiurus*.** Having an infamous reputation as the most dangerous genus of the Buthidae family, scorpions within the genus *Leiurus* are commonly found in NA, the Levant, and the Arabian Peninsula [93]. Although the *Leiurus* genus is currently divided into 12 species, it was once assumed to be represented by only a single species, *L*. *quinquestriatus* [83]. Therefore, most of the epidemiological data that were previously assigned to *L*. *quinquestriatus* must be reexamined, a situation which makes it very hard, if not impossible, to provide precise epidemiology data for different species of this genus [83]. Scorpions currently classified as *L*. *quinquestriatus* are restricted to Egypt and Sudan [109]. In upper Egypt, *L*. *quinquestriatus*, together with *A*. *crassicauda*, has been responsible for severe, even lethal envenomings. While the clinical manifestations were more severe in *L*. *quinquestriatus* stings, the death rate was higher in *A*. *crassicauda* stings [99]. Based on murine studies, it has been estimated that an injection of 17-mg *L*. *quinquestriatus* venom into an average-sized human adult might be lethal [110]. However, the average amount of venom injected in a sting is rather low (0.225 mg), which means that the risk of mortality is low in adults stung by *L*. *quinquestriatus* [111]. Nevertheless, the quantity of venom appears to be sufficient to pose a severe risk to children. For instance, it has been reported that *L*. *quinquestriatus* stings cause significant mortality among young children in Sudan [93]. The venom of *L*. *quinquestriatus* is extremely neurotoxic and may cause mild to severe local pain and severe systemic complications, such as vomiting, headache, disturbed consciousness, heart failure, pulmonary edema, interstitial edema, hemorrhage, and even coma [99,110,112]. In Jordan, *Leiurus hebraeus* and *Leiurus jordanensis* are considered to be dangerous to humans, with the former being the most common and widely distributed species in the country [113]. *L*. *hebraeus* stings are manifested by local pain of variable intensity at the site of the sting accompanied by mild swelling and tenderness. Still, systemic effects, such as vomiting, abdominal pain, sweating, salivation, tachycardia, hypertension, restlessness, tremors, and generalized myalgia, may evolve. In more severe cases, these symptoms are followed by agitation, hypotensive collapse with peripheral cyanosis, and signs of congestive cardiac failure that may simulate early myocardial infarction. Finally, interstitial myocarditis with local necrosis is seen in fatal cases [3].

***2*.*2*.*1*.*5*. *Mesobuthus*.** The *Mesobuthus* genus is the most widely distributed genus in the Buthidae family. It comprises 15 species dispersed across a variety of arid habitats of the Palearctic Region, from Turkey to Korea [114]. It has been reported that two species of this genus, i.e., *Mesobuthus gibbosus* and *Mesobuthus epeus*, can pose a risk of mild to severe envenoming to humans [115,116]. However, it is noteworthy that *M*. *eupeus* is a polytypic species complex that likely includes more than 1 valid species [117,118]. While *M*. *gibbosus* is mainly distributed in the eastern Mediterranean region [119], *M*. *epeus* has a wide geographical distribution in the ME and Central Asia. In the northwestern region of Iran, 80% of the 368 scorpion specimens collected between June 2015 and August 2016 belonged to *M*. *epeus* [120]. Although there are no data on scorpionism caused by *M*. *epeus* in this part of the country, it has been reported that 20% to 45% of all cases of scorpion stings in the Khuzestan Province (southwest) and Kashan (center) county, respectively, were caused by this species [121]. In Iran, stings by *M*. *epeus* are less dangerous than stings by *A*. *crassicauda* and *Hemiscorpius lepturus*, as so far, no deaths due to *M*. *epeus* stings have been reported [115,121]. However, *M*. *epeus* is still responsible for the majority of scorpionism cases in Iran [115]. It has been reported that local pain, hyperemia, swelling, and to a lesser extent, burning, itching, and numbness are seen among the victims. Moreover, dry mouth, thirst, sweating, hypotension, nausea, hypertension, difficulty in breathing, tachycardia, an increase of bronchial secretion, and cyanosis have also been reported [115]. It is believed that similar to other Buthids, the toxic effects of *M*. *epeus* are derived from various NTxs, including MeuKTx. This toxin specifically interacts with and blocks potassium ion channel subtype K_v_1.3 in humans [122]. K_v_1.3 is the first K^+^ channel that was identified outside electrically excitable tissues and its blockers have high significance as potential drugs against immune and inflammatory diseases [123].

#### 2.2.2. Hemiscorpiidae

***2*.*2*.*2*.*1*. *Hemiscorpius*.** Contrary to the widely distributed Buthidae family, scorpions belonging to the Hemiscorpiidae family are only found in Iran, Iraq, Pakistan, Saudi Arabia, Oman, UAE, and Yemen [124]. Additionally, this family is represented in Africa by three species, known only from a handful of specimens found in Egypt, Eritrea, and Somalia [125,126]. These three species all fall within the same and only Hemiscorpiidae genus, *Hemiscorpius*. Among 15 species within the *Hemiscorpius* genus, only two of them, i.e., *H*. *lepturus* and *Hemiscorpius acanthocercus*, have been reported to be medically important [127]. *H*. *lepturus* is considered to be responsible for almost all of the fatalities caused by *Hemiscorpius* stings, while only one case study has reported death due to *H*. *acanthocercus* envenoming [128]. However, it must be kept in mind that scorpions belonging to the *Hemiscorpius* genus look very similar in morphology, making it difficult for non-specialists to distinguish between different species [129]. Therefore, while there are many reports on the envenomings caused by *H*. *lepturus*, it is very likely that it is not the only species responsible for all of the cases [129–131].

Known as the only non-buthid scorpion that has the potential to be lethal to humans, *H*. *lepturus* is restricted to both sides of the common border between Iraq (southeast/east) and Iran (southwest), while stretching to the southern regions of the latter [118]. Annually, between 40,000 and 50,000 cases of scorpion stings (approximately 60 cases per 100,000 inhabitants) are recorded in Iran, resulting in approximately 20 fatalities [121]. It is estimated that *H*. *lepturus* is responsible for 10% to 25% of all sting cases and near 70% of the fatalities [121]. One possible explanation for the high lethality of *H*. *lepturus* stings is related to the small size of their stinger that is approximately 1 mm long (in comparison to 6 to 8 mm long stinger of *A*. *crassicauda*) [132,133]. It is believed that since the sting of *H*. *lepturus* does not penetrate the inner layers of skin, it causes no or minimal pain. This is the main reason why victims do not seek medical care until the toxicity has already been established. Another explanation focuses on the unique and exceptional venom composition of this species. While almost all of the medically important scorpion species from Buthidae and Scorpionidae families have neurotoxic effects, the venom of *H*. *lepturus* is more similar to the venom of *Loxosceles* spiders and has cytotoxic and hemotoxic effects (see Section 2.3.2.1) [129]. In the absence of pain-inducing NTxs, the symptoms of *H*. *lepturus* stings are largely negligible in the first 24 to 72 hours. After this period, however, local redness, inflammation, necrosis, ecchymosis, blisters, and hematuria may manifest, with the latter being one of the main reasons forcing victims to seek medical help [133]. Hemoglobinuria has been reported for more than half of *H*. *lepturus* sting cases (more than 90% of children present with this clinical manifestation), which, in turn, can be a sign of acute kidney injury that is frequently seen among the victims [134,135]. It has been suggested that the venom’s delayed effects and its retention in the body provides the toxins enough time to cause severe damage to vital organs, including the kidneys and, to a lesser extent, the liver. Death usually results from cardiovascular or renal failure [136]. However, even when victims survive, their hospitalization time is longer than that of victims stung by other scorpion species [137].

#### 2.2.3. Scorpionidae family

***2*.*2*.*3*.*1*. *Nebo*.** The *Nebo* genus is distributed in Egypt (Sinai), Israel, Jordan, Lebanon, Oman, Saudi Arabia, Syria, and Yemen [138]. The only medically important species of this genus, *Nebo hierichonticus*, is one of the least common but largest scorpions in Egypt, Israel, Saudi Arabia, and Syria [139]. With only two relatively old studies, the envenoming data for *N*. *hierichonticus* are very scarce. The first study (1969) reported only mild symptoms, such as mild local burning pain, swelling, and itching in two adults [140]. However, the second study reported the death of a child due to persistent hypotension, severe pulmonary edema, and congestive heart failure that was probably caused by toxic myocarditis. Other reported clinical manifestations included coagulopathy, temporary blindness, and deafness [141]. Together these studies indicate relatively mild venom toxicity that mostly poses a risk to children, elderly people, and victims suffering allergic reactions to the venom.

### 2.3. Spiders

**Fig 3:**
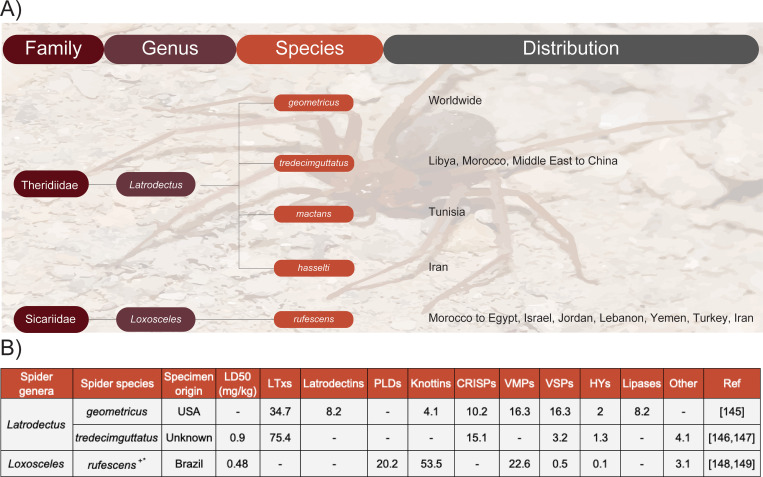
Medically relevant spiders in North Africa and the Middle East. **(A)** Phylogeny and geographic distribution and **(B)** reported toxicity and available venom proteome or transcriptome (^+^) data are shown [145–149]. Specifically, the table presents the genera, species, the origin of the reference proteome or transcriptome, and the median lethal dose in mice (LD_50_; mg/kg). It also illustrates the relative abundance of LTxs, Latrodectins, PLDs, ICKs (Knottins), CRiSPs, VMPs, VSPs, HYALs, and lipases. All components are represented as relative wet weight abundance in %. *proxy from *Loxosceles intermedia* was used. Notably, spider venom composition may vary depending on region. CRiSP, cysteine-rich secretory protein; HYAL, hyaluronidase; LTx, latrotoxin; PLD, phospholipase-D; VMP, venom metalloproteinase; VSP, venom serine protease.

It has been estimated that around five humans die from spider envenoming each year, with no death being reported from the MENA region [4]. The low number of fatalities despite the toxic venom of spiders is typically related to their small size, their docile nature, and the inability of their fangs to penetrate deeply into human skin [142]. Spider species with medical importance to humans are limited to six genera belonging to four different families; the genera *Atrax* and *Hadronyche* from the Atracidae family (Australian funnel-web spiders), the genus *Loxosceles* from the Sicariidae family (recluse spiders), the genus *Latrodectus* from the Theridiidae family (widow spiders), and the genus *Phoneutria* from the Ctenidae family (Brazilian armed spiders) [142]. Notably, Latrodectus and Loxosceles are both present in the MENA region and can cause adult morbidity and child mortality (Fig 3A).

In spiders, the synergistic effects between various toxins are particularly important for the whole venom toxicity [142]. Although proteomics or transcriptomic studies have only been conducted for the venoms of two out of the 10 potentially medically important spider species, it seems like *Latrodectus* venoms are predominantly made up of latrotoxins (34.7% to 75.4%), cysteine-rich secretory proteins (CRiSPs; 10.2% to 15.1%), and latrodectins (α-latrotoxin–associated low molecular weight proteins; 0% to 8.2%; Fig 3B). In contrast, *Loxosceles* venoms include inhibitory cysteine knot toxins (ICK peptides or Knottins; 53.5%), phospholipase-Ds (PLDs; 20.2%), and astacin-like metalloendopeptidases (MPs; 22.6%; Fig 3B) [142,143]. The synergy between spider toxins has also been demonstrated in a study that illustrated that *Loxoceles* sphingomyelinase Ds (SMDs) hydrolyze sphingomyelin (zwitterionic) to ceramide 1-phosphate (anionic); hence, they increase the affinity of NTxs for the nearby ion channels [144]. It appears that latrotoxins and PLDs are the most medically relevant toxins of the spiders found in the MENA region.

Below we present an overview of the medically relevant spiders in the MENA region and their most important toxins (Fig 3).

#### 2.3.1. Theridiidae

***2*.*3*.*1*.*1*. *Latrodectus*.** So far, 30 species of *Latrodectus* have been identified, inhabiting all continents, except for Antarctica [150]. Some of the most infamous species of this genus are *Latrodectus mactans* (North American black widow), *Latrodectus tredecimguttatus* (European black widow), *Latrodectus hasselti* (Australian red-back), and *Latrodectus geometricus* (cosmopolitan brown widow) [150–152]. These species are found within the MENA region, but while bites from members of these species can be fatal, no confirmed reports exist of *Latrodectus* induced mortality or morbidity in this region. The possible clinical manifestations caused by *Latrodectus* bites, known as latrodectism, include mild to moderate symptoms with generalized pain and muscle cramps being the most apparent. Typically, severe pain lasting for two days, on average, is observed in half of all *Latrodectus* bites. Furthermore, local effects such as redness at the bite site and swelling are also prevalent [153]. However, systemic effects such as nausea, vomiting, and headache are rare, and life-threatening symptoms generally only present in young children and elderly people [154].

Latrodectism is mainly induced by a neurotoxic component of the venom, i.e., ⍺-latrotoxin (⍺-LTx) [155]. *α*-LTx is a presynaptic NTx that affects the vertebrate central nervous system through depolarizing neurons by increasing Ca^2+^ concentration and inducing a massive release of neurotransmitters [146,156,157]. Proteomics data demonstrate that latrotoxins are the main components in the venoms of *L*. *tredecimguttatus* (75.4%) and *L*. *geometricus* (34.7%), yet little data are available on the abundance of latrotoxins in other spider species found in the MENA region (Fig 3B).

#### 2.3.2. Sicariidae family

***2*.*3*.*2*.*1*. *Loxosceles* genus.** Of about 120 described *Loxosceles* species, *Loxosceles rufescens* is the only species that has been reported to be of medical importance in the MENA region. *L*. *rufescens* (Mediterranean recluse) is found from Morocco to Egypt, as well as in Iran, Israel, Jordan, Lebanon, and Yemen [143]. In Iran, *L*. *rufescens* is widely distributed across the country [158–161]. Envenoming by these spiders can cause necrotizing-hemolytic effects, known as loxoscelism. Loxoscelism is commonly associated with skin lesions, while systemic effects such as nausea, vomiting, chills, fever, myalgia, hemolytic anemia, and acute renal failure are rarely observed [161]. In Iran, loxoscelism can be misdiagnosed as *Hemiscorpius* scorpionism, since both *Loxosceles* spiders and *Hemiscorpius* scorpions contain SMD, a necrotic toxin, in their venom [162].

SMD is a member of phospholipase D protein family that makes up to approximately 20% of *Loxosceles* venoms (Fig 3B) [163–165]. PLDs are the most studied toxic compounds of the *Loxosceles* venom and have hydrolyzing activity on the D site of phospholipids in various mammalian tissues [129]. Cleavage at the D site of phospholipids is rare and has not been identified elsewhere in the animal kingdom other than for PLDs from *Loxosceles* spiders and *Hemiscorpius* scorpions [153]. Notably, the primary causative agent in necrotic lesion formation in *Loxosceles* spider and *Hemiscorpius* scorpion envenomings are a group of PLDs and SMDs [163–165]. In addition to the dermonecrotic characteristics, PLDs are responsible for various pathologies ascribed to the whole venom of *Loxosceles* spiders, including an inflammatory response accompanied by neutrophil infiltration and complement activation, increased permeability of the endothelial lining of blood vessels, hemolysis, platelet aggregation, and renal failure. This venom toxin induces cell lysis by hydrolysis of sphingomyelin and complement-dependent hemolysis [166]. Unfortunately, the process in which the cleavage of sphingomyelin leads to necrotic lesion formation is poorly understood, although a complex immune response has been indicated as a primary instigator [167].

## 3. Animal envenomings in the MENA region: Country-specific impact

**Fig 4:**
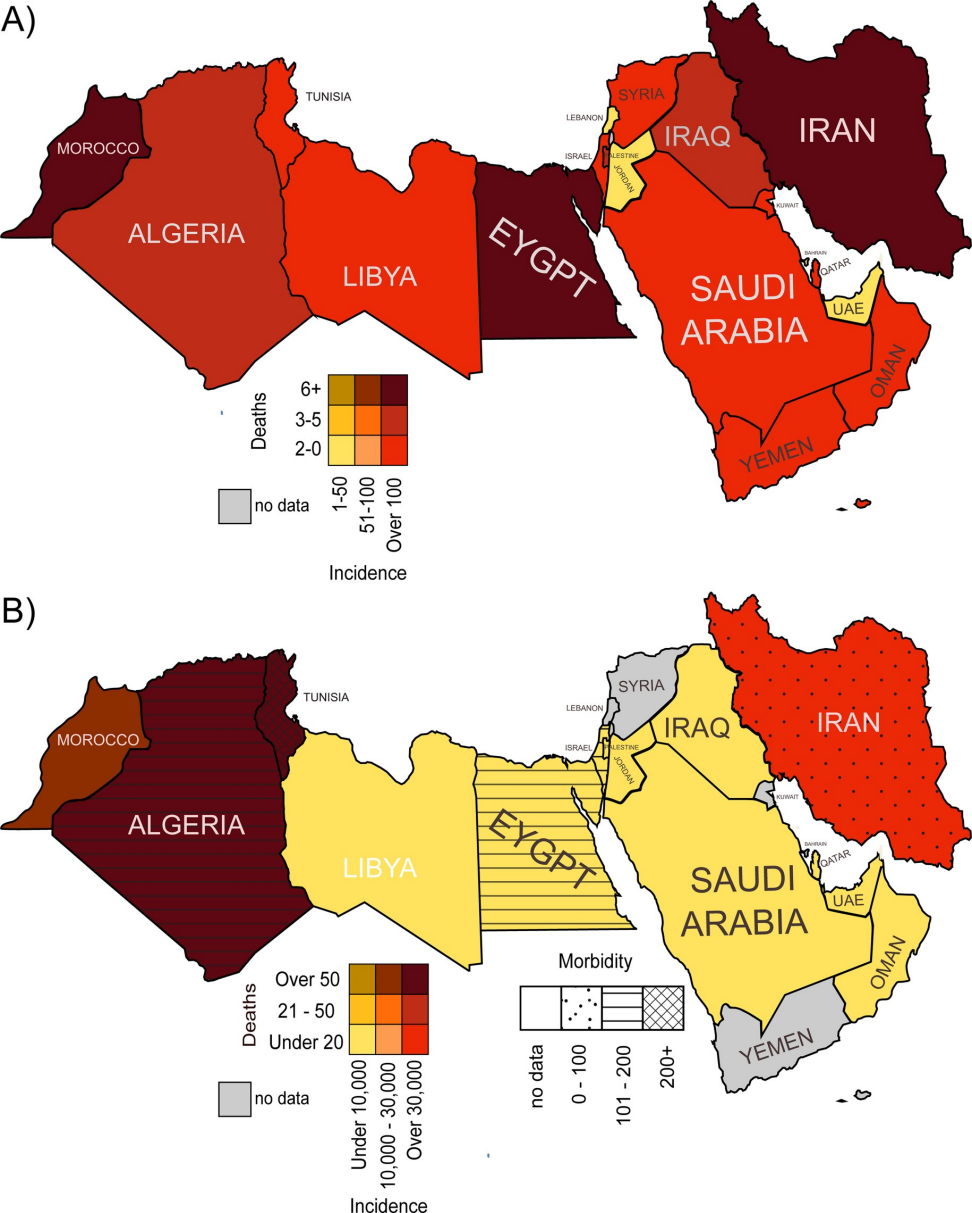
Country-specific overview of the epidemiology of snakebite (A) and scorpion stings (B) in the MENA region. The country-specific data were obtained from epidemiological literature covering the past 25 years (1995 to 2020). The data represent absolute numbers, and details can be found in S1 Table. MENA, Middle East and North Africa.

Estimates suggest that NA records over 36,000 snakebites (29 deaths) and 350,0000 scorpion stings (810 deaths) and the ME more than 31,000 snakebites (33 deaths) and 146,000 scorpion stings (796 deaths) each year [2,17]. However, actual country-specific epidemiological reports are scarce and fragmented, particularly for snakebite. Therefore, the following should not be seen as a comprehensive overview, but rather an indication as to where potential envenoming hotspots might be and thus, where to focus further research efforts (Fig 4 and S1 Table) [2,17–31,77,78,80,99,113,121,127,168–186].

Most snakebites in the MENA region occur in rural, aridic regions during the summer months and in the evening hours, with some data suggesting that primarily men are bitten. In the ME, patients are typically young and in most cases below the age of 30 (S1 Table). According to the literature published over the past 25 years, Morrocco and Egypt suffer from most snakebites in NA, with annual incidences of 137 and 246, respectively (Fig 4A) [17,23,25]. However, these numbers are still relatively low when compared to prior estimates that were made based on a literature review and a modeling study (up to 6,349 and 21,245 bites per year, respectively) [17]. In the ME, Iran (5,379), followed by Israel (322), presents the highest incidence of snakebite (Fig 4A) [18,21,138,139]. Notably, estimates suggested that up to 11,079 bites occur in Iran each year (significantly more than reported), while Israel’s estimates are lower than what is reported in the literature (155) [17]. The presence or absence of such discrepancies are often a good indication of whether snakebite is reported appropriately and comprehensively in the respective country. When there are significant differences between published data and estimates, usually estimates represent the true burden better since published data are frequently incomplete [17]. Based on epidemiological estimates, Yemen (7,949), Syria (4,651), and Iraq (4,366) should also receive further attention [17]. Morbidity data are also lacking, which in other snakebite affected regions often constitutes the majority of the overall health burden, as well as mortality data [7].

While the issue of snakebite in the MENA region should not be neglected, scorpionism constitutes a significantly larger problem. The majority of stings occur in the summer months, equally affecting men and women, with primarily children being stung (S1 Table). In NA, Algeria [187–189] and Tunisia [78] both report over 40,000 stings from scorpions each year, resulting in 73 and 10 annual deaths, respectively (Fig 4B). Furthermore, although Morocco reports fewer annual incidences than Algeria and Tunisia (21,467), it has higher annual deaths (86) [80]. This, in part, could be due to the fact that Morocco is inhabited by 12 different species of highly venomous Buthidae scorpions [80]. In terms of morbidity, Tunisia seems to be most affected in the whole MENA region, with over 425 cases recorded each year. In the ME, Iran (50,000) [121,127,172] and Saudi Arabia (14,433) [173] report the highest number of annual scorpion stings. However, similarly to the snake epidemiological data, these numbers still appear to be very low when compared to the comprehensive estimations mentioned above.

## 4. Treatment of severe animal envenomings in the MENA region

To date, the only effective treatment for envenoming consists of serotherapy [190]. Here, immunity is induced in a production animal (e.g., horse or sheep), and the resultant polyclonal antibodies isolated from the hyperimmunized plasma are transfused into the patient. Thus, it is also known as passive vaccination [7]. A vital advantage of this approach is that in the case of highly diverse toxin cocktails that are commonly found in animal venoms, it is not essential to know what specific toxins are present, as long as the immune system of the production animal gives rise to neutralizing antibodies against all the key toxic components [190]. Although serotherapy has been the mainstay in treatment of envenomings for over a century and has saved countless lives, the complexity and high cost of production, the decreasing number of antivenom products, and the fragility of the production systems in developing countries contribute to the risk of not having effective, safe, and affordable antivenoms in the future. Therefore, there is a global push toward development of improved envenoming therapies [191]. Efforts include the improvement of existing antivenoms via better immunization and antibody purification approaches [190,192], as well as the discovery of specific antibodies and alternative protein scaffolds that target specific toxins, among others [193]. Furthermore, small-molecule inhibitors that target phospholipase A_2_s (varespladib), snake venom metalloproteinases (marimastat, batimastat, dimercaprol, and 2,3-dimercaptopropane-1-sulfonic acid), and snake venom serine proteinases (nafamastat) are particularly interesting in the context of envenomings in the MENA region [8–14].

### 4.1. Current treatments

Until novel types of antivenom products reach the market, prompt administration of the appropriate polyvalent antivenom in the approved dose is the best treatment following an envenoming by a venomous animal [194]. Currently, eight snake, 11 scorpion, and two spider antivenoms exist that, according to the manufacturers, cover medically relevant species that inhabit the MENA region [2,3]. All of the antivenoms are based either on either immunoglobulin G antibodies (IgGs) or are F(ab’)_2_ fragments that are generated by pepsin digestion of whole IgG antibodies to remove most of the fragment crystallizable (Fc) region (i.e., the tail region of an IgG antibody that interacts with cell surface receptors), while leaving intact some of the hinge region. Thus, like IgG antibodies, F(ab’)_2_ fragments have two antigen-binding F(ab) portions linked together by disulfide bonds, and therefore are divalent.

#### 4.1.1. Snake antivenoms

Currently, there are 10 antivenoms marketed as efficacious against snake species found across the MENA (Table 1). A lack of clinical data necessitates the assessment of a wide range of efficacy evidence, including preclinical investigations, observational clinical studies, and expert opinions from clinicians with extensive experience managing African snakebites; on that basis, SAIMR Polyvalent, Inoserp MENA, and NAVPC Polyvalent Snake Antivenom are generally considered to be relatively safe and effective [195]. Notably, Inoserp MENA are lyophilized products, allowing them to be stored at ambient tropical temperatures and injected intravenously, allowing for use in poorly equipped and/or rural health centers. Conversely, while considered one of the most effective antivenoms SAIMR must be refrigerated; in addition, it is known to cause a high rate of early adverse reactions and is very expensive [196,197]. Indeed, SAIMR Polyvalent antivenom, which currently incorporates venoms from 10 species (two vipers and eight elapids) is sold at a cost of approximately $117 USD per vial. Typically, a patient will require 6 to 10 vials for successful treatment resulting in a cost of more than $600 USD for only the antivenom [198]. Thus, the resultant increased demand for cheaper antivenoms has already resulted in the sale and use of products developed for similar species from Asia, but with potentially dangerously low efficacy and no proven cross-reactivity [199,200]. For instance, at the main government hospital in Taizz (Yemen), the only antivenom in use was the imported Indian Snake Venom Antiserum I.P. VINS Bioproducts; this product covers the Indian cobra (*Naja naja*), the common Krait (*Bungarus caeruleus*), the Russell’s viper (*Daboia russelii*), and the saw scaled Viper (*E*. *carinatus*). None of these species can be found in Yemen, but the hope for paraspecificity stems from the fact that other species from some of the same genera inhabit Yemen. Unfortunately, evidence to date suggests poor efficacy at best [199,200]. Some data suggest that Anti-*Daboia palaestinae* monospecific antivenom (Felsenstein Medical Research Center) [201], Gamma-Vip (Institut Pasteur de Tunis) [43], and the Penta/Hexavalent Snake Antivenom IgG (Razi) [202] should be efficacious, while no reliable efficacy data exist for VACSERA polyvalent antivenom, Anti-vipérin (Institut Pasteur d’Algerie) [196].

**Table 1:** Snake antivenoms currently available for use in MENA.

Antivenom name	Producer	Species venoms neutralized according to manufacturer	
		Viperidae	Elapidae
INOSERP MENA	Inosan Biopharma, **Spain**	*B*. *arietans*, *C*. *cerastes*, *C*. *gasperettii*, *C*. *vipera*, *Daboia deserti*, *D*. *mauritanica*, *D*. *palaestinae*, *E*. *carinatus sochureki*, *E*. *coloratus*, *E*. *khosatzkii*, *E*. *leucogaster*, *E*. *megalocephalus*, *E*. *omanensis*, *E*. *pyramidum*, *M*. *lebetina*, *M*. *Iebetina transmediterranea*, *M*. *Iebetina turanica*, *Montivipera bornmuelleri*, *M*. *raddei kurdistanica*, *P*. *fieldi*, *P*. *persicus*, *Vipera latastei*	*N*. *haje*, *N*. *nubiae*, *N*. *pallida*, *Walterinnesia aegyptia*
SAIMR Polyvalent Antivenom	South African Vaccine Producers (SAVP) **South Africa**	*B*. *arietans*, *B*. *gabonica*	*Hemachatus haemachatus*, *Dendroaspis angusticeps*, *D*. *jamesoni*, *D*. *polylepis*, *N*. *nivea*, *N*. *melanoleuca*, *N*. *annulifera*, *N*. *mossambica*
Polyvalent Snake Antivenom	National Antivenom and Vaccine Production Center (NAVPC), **Saudi Arabia**	*B*. *arietans*, *E*. *coloratus*, *E*. *carinatus*, *C*. *cerastes*,	*N*. *haje (N*. *arabica)*, *W*. *aegyptia*
Bivalent Snake Antivenom			*N*. *haje (N*. *arabica)*, *W*. *aegyptia*
VACSERA polyvalent antivenom	Egyptian Organization for Biological Products & Vaccines, **Egypt**	*C*. *cerastes*, *C*. *cerastes cerastes*, *C*. *vipera*, *P*. *persicus fieldi*	*N*. *haje*, *N*. *nigricollis*, *N*. *pallida*, *W*. *aegyptia*
Anti-vipérin	Institut Pasteur d’Algerie, **Algeria**	*C*. *cerastes*, *M*. *lebetina* spp.	
Anti-*D*. *palaestinae* monospecific antivenom	Felsenstein Medical Research Center, **Israel**	*D*. *palaestinae*	
Gamma-Vip	Institut Pasteur de Tunis, **Tunisia**	*C*. *cerastes*, *M*. *lebetina* spp.	
Pentavalent Snake Antivenom IgG	Razi Vaccine and Serum Research Institute, **Iran**	*V*. *lebetina*, *V*. *albicornuta*, *E*. *carinatus*, *P*. *persicus*, *Agkistrodon halys*	
Hexavalent Snake Antivenom IgG		*V*. *lebetina*, *V*. *albicornuta*, *E*. *carinatus*, *P*. *persicus*, *A*. *halys*	*N*. *oxiana*

All listed antivenoms are produced as F(ab’)_2_s.

IgG, immunoglobulin G; MENA, Middle East and North Africa.

There is a need for more cost-effective antivenoms in the MENA region as well as the need to keep future treatments at a low cost (affordable to the general population) while retaining efficacy.

#### 4.1.2. Scorpion antivenoms

Currently, 11 antivenoms to treat scorpionism exist in the MENA region (Table 2), yet the treatment of scorpion envenomings with antivenom remains debated. This might, in part, be due to inconclusive clinical trial outcomes, with studies focusing on scorpionism in the MENA region yielding conflicting results. For instance, a study in Tunisia with 875 patients showed no benefits of using bivalent antivenom (produced by Institut Pasteur de Tunis) after envenoming by what was assumed to be *A*. *australis* and *B*. *occitanus* scorpions [203]. Yet, it is noteworthy that the trial did not include a lot of cases of severe or systemic envenoming (145 patients with systemic symptoms of whom 11 had life-threatening signs), where antivenom might have had a bigger impact on clinical outcome. In comparison, a retrospective Moroccan trial with 275 patients (28 with systemic symptoms) investigated the specific use of rapid administration of a polyvalent scorpion antivenom (produced at Institut Pasteur du Maroc) following envenoming by *A*. *mauretanicus* and *B*. *occitanus* scorpions. The use of this antivenom was associate with a decrease in envenoming clinical signs in patients who received prompt and specific antivenoms [204]. Additionally, a study by the French military demonstrated that 19 patients were effectively treated with SCORPIFAV (MicroPharm) [205]. There also exists some preclinical data indicating the potential usefulness of antivenoms in treating scorpionism for Polyvalent Scorpion Antivenom (Razi) [206–209], Purified polyvalent anti-scorpion serum (VACSERA) [210], Inoscorpi (Inosan Biopharma) [211]. No data could be found for Polyvalent scorpion antivenom (Institut Pasteur du Maroc), Anti-scorpionique (Institut Pasteur d’Algerie), Anti-scorpionic sera (Institut Pasteur de Tunis) Polyvalent scorpion antivenom (NAVPC), and SAIMR Scorpion antivenom; the same is true for Scorpion Venom Antiserum (VINS), with efficacy being unlikely due to the reliance on paraspecificity.

**Table 2:** Scorpion antivenoms currently available for use in the MENA.

Antivenom name	Producer	Species venoms neutralized according to product insert
Polyvalent scorpion antivenom	Institut Pasteur du Maroc, **Morocco**	*B*. *occitanus*, *Androctonus mauritanicus*
Purified polyvalent anti-scorpion serum	Egyptian Organization for Biological Products & Vaccines (VACSERA), **Egypt**	*A*. *amoreuxi*, *A*. *australis*, *A*. *bicolor*, *A*. *crassicauda*, *B*. *occitanus*, *Leiurus quinquestriatus*, *Scorpio maurus*
Anti-scorpionique (Monovalent)	Institut Pasteur d’Algerie, **Algeria**	*A*. *australis*
Bivalent scorpion antivenom	Institut Pasteur de Tunis, **Tunisia**	*A*. *australis*, *B*. *occitanus*
Anti-scorpionic sera		*A*. *australis*, *A*. *bicolor*, *B*. *occitanus*, *L*. *quinquestriatus*
Polyvalent scorpion antivenom	National Antivenom and Vaccine Production Center (NAVPC), **Saudi Arabia**	*L*. *quinquestriatus*, *A*. *crassicauda*, *Buthacus arenicola*, *Hottentotta minax*, *B*. *occitanus*, *L*. *hebraeus and A*. *amoreuxi*
SCORPIFAV	MicroPharm, **UK**	*A*. *australis*, *B*. *occitanus*, *and L*. *quinquestriatus*
Scorpion Venom Antiserum (VINS)	VINS Bioproducts, **India**	*Specific for L*. *quinquestriatus and A*. *ameoreuxi* *Paraspecific for A*. *crassicauda*, *A*. *aeneas*, *A*. *australis*, *S*. *marus [Sic] palmatus*, *and Bathus [Sic] occitanus*
Polyvalent Scorpion Antivenom	Razi Vaccine and Serum Research Institute, **Iran**	*A*. *crassicauda*, *M*. *eupeus*, *O*. *doriae*, *H*. *saulcyi*, *H*. *schach and H*. *lepturus*
Middle East & Northern Africa (MENA) Inoscorpi High Specificity Immunotherapic Polyvalent F(ab′)_2_ Scorpion Antivenom lyophilized	Inosan Biopharma, **Spain and Mexico**	*A*. *australis*, *A*. *mauritanicus*, *A*. *crassicauda*, *A*. *amourexi*, *A*. *bicolor*, *B*. *occitanus*, *B*. *mardochei*, *L*. *quinquestriatus*, *L*. *hebraeus*, *B*. *tunetanus*
SAIMR Scorpion antivenom	South African Vaccine Producers, **South Africa**	*Parabuthus* sp.

Most of the listed antivenoms are produced as F(ab’)_2_s, with the exception of Scorpion Venom Antiserum (VINS) and Polyvalent scorpion antivenom (NAVPC), which are IgGs.

IgG, immunoglobulin G; MENA, Middle East and North Africa.

Skepticism against the use of antivenoms to treat scorpionism also stems from their questionable cost-effectiveness, given that the treatment can be rather expensive [212]. Fortunately, unlike in the United States of America where a single vial of scorpion antivenom can cost around $3,500 USD, there are significantly cheaper products available in the MENA region. For instance, the bivalent scorpion antivenom (produced at Institut Pasteur de Tunis) costs approximately $13 USD per vial, which when taking local economics into account is not cheap, but significantly more affordable [213]. Due to the lower cost, 6,226 to 7,253 doses of just this bivalent scorpion antivenom are sold each year (2016 to 2018). Thus, when taking in to account the improved price-point of scorpion antivenom in the MENA region compared to the USA, as well as investigations of antivenom treatment of scorpionism outside of the MENA region, it is likely that antivenom can be cost-effective and useful in most, and necessary in severe, envenoming cases [214]. However, the question remains whether currently available antivenoms are able to sufficiently neutralize toxicity across all medically relevant scorpion species in the MENA region.

#### 4.1.3. Spider antivenoms

While spider bites do not constitute a substantial medical threat in the MENA region, this might change in the near future. This is due to the fact that many of the species, such as *L*. *hasselti* and *L*. *mactans*, are invasive and will potentially spread in the years to come. Fortunately, two antivenoms from the countries these species are native to (Australia and the USA) already exist (Table 3). In Brazil, Peru, and Mexico, there are also anti-*Loxosceles* antivenoms being produced, but none of them specifically use *L*. *rufescens* venom in their immunizations [215]. Regardless, similarly to antivenom treatment in cases of scorpionism, the use of antivenom treatment in spider envenomings has also been debated. The key criticisms are also comparable to scorpion antivenom and involve the discussion of their efficacy in neutralizing toxicity, as well as the risk of adverse reactions [216,217]. Nevertheless, the overall conclusion remains the same as in the case of scorpion stings; in cases of severe envenoming, antivenoms can significantly benefit patient outcome and can be necessary in the prevention of morbidity and mortality of the victim [215,216]. Unfortunately, the manufacture of spider antivenoms is significantly more cumbersome than similar products against scorpions and snakes, since the venom amounts required for immunization are very hard to obtain and the actual spider “milking” process is incredibly laborious [218]. Indeed, sufficient numbers of specimens for venom extraction are a key bottleneck in spider antivenom production. For instance, in 2015 and 2017, the Australian Reptile Park that is responsible for collecting and milking venom from male *Atrax robustus* spiders used for antivenom production, urged the public to catch Sydney funnel-web spiders to secure a continuous supply of antivenom [219,220]. However, novel approaches in antivenom manufacture, such as the recombinant expression of toxins for immunization [192] or RNA vaccination [206], could help circumvent such bottlenecks and help ensure a steady supply of antivenoms.

**Table 3:** Spider antivenoms currently available for use in MENA.

Antivenom name	Producer	Species venoms neutralized according to product insert
Red Back Spider Antivenom	Sequirus, **Australia**	*L*. *hasselti*
Antivenin *Latrodectus mactans*	Merck & Co, **USA**	*L*. *mactans*

All of the listed antivenoms are produced as IgGs.

IgG, immunoglobulin G; MENA, Middle East and North Africa.

### 4.2. Perspectives for future treatments

While antivenoms currently constitute the only effective treatment for severe animal envenomings, these life-saving medicines also suffer from many drawbacks. Thus, researchers are investigating other avenues of treatment, such as the targeted neutralization of key medically relevant toxins using recombinant monoclonal antibodies [193,218,221,222]. In particular, two major approaches toward the discovery and development of such antibodies exist, i.e., “top-down” and “bottom-up”. The former involves immunization of an animal, from which polyclonal antibody-encoding genes are derived and used for recombinant production of the antivenom [223]. Here, the development of the final product might be less costly, but the product is of undefined polyclonal nature and relatively expensive to manufacture, as it will comprise both therapeutically active and inactive antibodies, resulting in higher doses being needed for venom neutralization. Conversely, the bottom-up strategy uses in-depth knowledge on snake venom compositions (e.g., via toxicovenomics [224]), which allows for the strategic selection of a defined number of well-characterized monoclonal antibodies via *in vitro* display technologies, such as phage, ribosome, mammalian, or yeast display. The selected antibodies can then be mixed in a defined cocktail, constituting an oligoclonal recombinant antivenom [12]. These technologies can aid in the discovery of broadly neutralizing antibodies, especially when combined with other approaches, such as toxin cross-panning (i.e., different toxins are alternated during selection rounds in a display campaign) [225] or the use of consensus toxins (*in silico* design of a “consensus” toxin sequence for a set of target toxins) [226,227]; discovery of such broadly neutralizing antibodies will help minimize the total number of antibodies required for a polyvalent recombinant antivenom and thus significantly reduce the cost of the final product [223]. The hope is that these approaches will help lead to the development of antivenoms with superior therapeutic properties (efficacy, safety, and quality) [6] composed by a low number and volume of antibodies (as all included antibodies will be broadly neutralizing and therapeutically relevant), allowing for lower cost of manufacture than the polyclonal recombinant antivenoms [198,228]. However, oligoclonal recombinant antivenoms have longer development timelines and therefore come with a higher R&D cost [229]. Besides traditional antibodies, other scaffolds, such as nanobodies [230], are also proving promising in the treatment of envenomings [193].

Based on the venom proteomes available for snakes, scorpions, and spiders inhabiting the MENA region, the development of recombinant antivenoms should particularly focus on the discovery of monoclonal antibodies against SVMPs, SVSPs, 3FTxs, and PLA_2_s to treat snakebite, against NaTxs and KTxs for the treatment of scorpionism, and against latrotoxins and PLDs for recombinant spider antivenoms. Notably, a recombinant monoclonal antibody against α-Latrotoxin from the Mediterranean Black Widow Spider (*L*. *tredecimguttatus*) already exists, which has been shown to bind and neutralize this toxin *ex vivo* [231]. Supported by the comparatively small number of different toxins that require neutralization, the costly production of existing conventional antivenoms, and challenging maintenance of the venom supply chain, it thus is possible that the implementation of such recombinant therapeutics might first occur in spiders, likely followed by scorpions, and only then venomous snakes (due to the increasing complexity of their venoms).

The development of next-generation antivenoms is, however, not only driven by the discovery of recombinant toxin-targeting antibodies, but also by the repurposing of existing and sometimes even approved drugs, such as small molecule inhibitors and metal ion chelators that target phospholipase A_2_s (varespladib) and snake venom metalloproteases (e.g., marimastat, dimercaprol, and DMPS). Indeed, initial findings have demonstrated that varespladib neutralized, *in vitro*, the vast majority of anticoagulant activities and even some procoagulant activities present across a range of snake venoms, of which some are from MENA, (i.e., *E*. *carinatus*, *E*. *ocellatus*, *D*. *russelii*, *B*. *arietans*, *Bothrops asper*, *Bothrops jararaca*, *Calloselasma rhodostoma*, and *Deinagkistrodon acutus*) [232,233]. Furthermore, a study has demonstrated that varespladib can abrogate or delay lethality induced by presynaptically acting neurotoxic snake venom in murine models; this might in part be due to the inhibition of PLA_2_-induced anticoagulation preventing the rapid spread of the NTxs [9]. Additionally, marimastat demonstrated impressive *in vitro* neutralization of procoagulant activities in most of these venoms, while dimercaprol and DMPS were only able to partially neutralize these activities [232,233]. Together, marimastat and varespladib were even able to prevent murine lethality caused by venom from the most medically important vipers of Africa, South Asia, and Central America [13]. Due to the differences in the venom toxin families present in snakes when compared to scorpions and spiders, these small molecules have little application in the latter two animal groups.

Combined, recombinant antibody technologies and the repurposing of small molecule inhibitors give hope toward the possibility that in the future, significantly improved and cost-effective antivenom products will become available for the MENA region [8–14].

## 5. Outlook

Animal envenomings in the MENA region constitute a significant health burden that deserves further attention. Indeed, given the number of people affected by these neglected diseases, it is pivotal to ensure that effective and affordable treatments are readily available. Antivenoms currently available in the region are vital in preventing the health impact from worsening, yet ample room for innovation remains. While challenged with a diverse array of toxin targets, recombinant antivenoms, consisting of defined, toxin-targeting cocktails of monoclonal antibodies, present an interesting opportunity to potentially improve future envenoming therapy in the MENA region. However, to enable the effective development of new treatments, it is pivotal to address existing gaps in epidemiological data. This will help ensure that discovery approaches are focused on the most affected areas and that scientific endeavors are aligned with actual medical needs. Furthermore, a comprehensive analysis of the venoms of all medically relevant venomous species will need to precede any such efforts, since only a fraction of the required venom proteomes is available. Nevertheless, these challenges do not present unsurmountable obstacles, but rather scientific milestones to aim for. In the future, improved and affordable envenoming treatments will hopefully become a reality for patients in the MENA region and beyond.

## 6. Methods

We searched for articles indexed in PubMed until February 23, 2021 using the following keywords: snake*, scorpion*, spider*, North Africa, Middle East, MENA, toxin, venom, omic, sting, bite, antiven*, envenom*, epidemiology, and treatment. This search was followed by a snowball search, manually reviewing literature reviews, and using Google Scholar/Researchgate. All the original research or review articles in either English, Persian, or Arabic that presented data directly relevant to animal envenomings in the MENA region or provided supporting information were eligible. We obtained close to 1,000 references, and which after eliminating duplicates and excluding articles that did not match the eligibility criteria, 211 of them were selected. The relevance of the epidemiological references was further assessed while a primary focus was placed on including data from the past 25 years (i.e., 1995 to 2020). From the final 59 epidemiology related references that we selected, 33 were retrospective, nine were reviews, eight were case reports, six were prospective, and three were cross-sectional studies. The variables extracted were publication identifiers (authors, journal, and year of publication); study characteristics (study design, period, country, sample size, and age groups); taxonomy of the venomous animal (species); and demographics, locality, season/time, anatomical site of bite/sting, symptoms, and the undertaken management approaches (c.f., S2 Table). In general, wherever possible, the incidences (i.e., the number of venomous animal encounters in a given period of time, disregarding the severity of symptoms) were presented as per 100,000 people per year. To supplement the scarce information on antivenoms for the MENA region available from the literature, antivenom producers were contacted. Unfortunately, only a few responded to enquiries (i.e., Inosan, Razi, and SAVP); the provided information was implemented wherever relevant.

**Key Learning Points**

- In the Middle East and North Africa (MENA) region, scorpion stings are significantly more common (approximately 350,000 cases/year) than snakebites (approximately 70,000 cases/year) and present the most significant contributor to overall health burden of enevenomings.
- There is a substantial lack of high-quality envenoming data available for the MENA region.
- In the MENA region, the majority of snake venoms contain snake venom metalloproteinases, while sodium channel–binding toxins and potassium channel–binding toxins are the scorpion toxins that cause most health-related challenges.
- There are a series of different antivenoms for envenoming in the MENA region, but only few are clinically validated, and their high cost and limited availability present a substantial health challenge.
- Future therapeutic solutions, such as next-generation antivenoms based on monoclonal antibodies and/or small molecule inhibitors, might help address this issue.

**Top Five Papers**

1. Amr ZS, Baker MAA, Warrell DA. Terrestrial venomous snakes and snakebites in the Arab countries of the Middle East. *Toxicon*. 2020;177:1–15.
2. Amr ZS, Abu Baker MA, Al-Saraireh M, Warrell DA. Scorpions and scorpion sting envenoming (scorpionism) in the Arab Countries of the Middle East. *Toxicon*. 2021;191:83–103. doi: 10.1016/j.toxicon.2020.12.017
3. Kasturiratne A, Wickremasinghe AR, de Silva N, Gunawardena NK, Pathmeswaran A, Premaratna R, et al. The global burden of snakebite: a literature analysis and modelling based on regional estimates of envenoming and deaths. *PLoS Med*. 2008;5:e218. doi: 10.1371/journal.pmed.0050218
4. Chippaux J-P, Goyffon M. Epidemiology of scorpionism: A global appraisal. *Acta Trop*. 2008;107:71–79. doi: 10.1016/j.actatropica.2008.05.021
5. Gutiérrez JM, Calvete JJ, Habib AG, Harrison RA, Williams DJ, Warrell DA. Snakebite envenoming. *Nat Rev Dis Primer*. 2017;3:17063. doi: 10.1038/nrdp.2017.63

## Supporting information

S1 Table Overview of the epidemiology of snakebites and scorpion stings in the MENA region. The population data for the 5 North African and 14 Middle Eastern countries as well as their allocation in “urban” and “rural” was obtained from the world development indicators database (The World Bank). The information about incidences, antivenom administration, locality of incidence, age groups, gender ratios, morbidity, and mortality were retrieved from articles published in scientific journals. The study type and the geographical region covered are also included. Due to the regional limitations of most articles, countrywide numbers were either estimated by us based on the literature of several different regions that cover the major topology of a country and are geographically evenly distributed (light green), or we used prior population adjusted (increased population since publication) estimates from Kasturiratne and colleagues for snakebites and Chippaux and Goyffon for scorpion stings (yellow), if there was insufficient literature. However, some articles gave a clear indication that the numbers are covering the epidemiology of the entire country (dark green). Relevant publications are included in brackets. MENA, Middle East and North Africa.
(XLSX)

S2 Table Overview of epidemiological studies included in this review. Parameters assessed included study design, country that the study was conducted in, the study period, how many patients were included (*N*), the respective patient age groups, and what venomous taxa were responsible for the envenomings. It also states whether the articles contained information on the demographics, locality, season/time, species, anatomical site of bite/sting, symptoms, and management.
(XLSX)
